# Infections Caused by Carbapenemase-Producing *Klebsiella pneumoniae*: Microbiological Characteristics and Risk Factors

**DOI:** 10.1089/mdr.2018.0339

**Published:** 2019-03-08

**Authors:** Hongying Pan, Yaling Lou, Linyan Zeng, Li Wang, Jiajie Zhang, Wei Yu, Yunqing Qiu

**Affiliations:** ^1^ Department of Infectious Diseases, Zhejiang Provincial People's Hospital, People's Hospital of Hangzhou Medical College, Hangzhou, China.; ^2^ State Key Laboratory for Diagnosis and Treatment of Infectious Disease, Collaborative Innovation Center for Diagnosis and Treatment of Infectious Diseases, The First Affiliated Hospital, College of Medicine, Zhejiang University, Hangzhou, China.; ^3^ Department of Infectious Diseases, Dongyang People's Hospital, Jinhua, China.; ^4^ Department of Intensive Care Unit, The First Affiliated Hospital, College of Medicine, Zhejiang University, Hangzhou, China.

**Keywords:** carbapenemase-producing *K. pneumoniae*, risk factors, mortality, hypervirulent phenotype, cost

## Abstract

The spread of carbapenemase-producing *Klebsiella pneumoniae* (CPKP) worldwide is a serious problem. This retrospective, matched case–control, parallel study in a tertiary teaching hospital analyzed the microbiological and clinical characteristics of CPKP infection, focusing on the risk factors for carbapenem resistance and patient mortality. The hospital department with the highest incidence of CPKP infections was the intensive care unit. All CPKP strains examined were positive for *bla_kpc-2_*, and 84.8% of CPKP were ST11. Hypervirulent phenotype was identified in 22.7% of the patients with CPKP, with these strains displaying a high incidence of positivity for *entB*, *ybtS*, and *iutA*. Multivariate conditional logistic regression analysis demonstrated that Pitt bacteremia score >4, prior stomach tube, continuous renal replacement therapy (CRRT), and previous carbapenem exposure were associated with CPKP infection. Higher albumin concentration and use of cephalosporins after diagnosis were strong prognostic factors for crude 28-day mortality. Further, high APACHE II score, CRRT, use of carbapenems after diagnosis, and bacteremia were risk factors for crude in-hospital mortality. CPKP isolates showed clonal spread and were resistant to most antibiotics, resulting in higher financial burden. Critical illness was associated with increased mortality.

## Introduction

*K**lebsiella pneumoniae* are normal microbiota that can colonize the upper respiratory tract, gastrointestinal tract, and urinary tract in humans.^1^ Carbapenem antibiotics were regarded as reliable and potent agents against *K. pneumoniae.*^2^ However, over the past 10 years, the worldwide incidence of carbapenemase-producing *K. pneumoniae* (CPKP) has increased dramatically, posing a serious threat to patients.^3^ Between 2005 and 2014 in China, *K. pneumoniae* strains resistant to imipenem and meropenem have increased from 2.4% to 10.5% and from 2.6% to 13.4%, respectively.^4^

Infections with CPKP are difficult to control due to the spread of carbapenem-resistant genes via transferable plasmids.^5^ Treatment options in patients infected with CPKP are also limited, and few clinical studies have recommended appropriate antibiotics.^6^ Although colistin, fosfomycin, tigecycline, and selected aminoglycosides were considered effective in treating CPKP infections,^6^ they were unable to eradicate CPKP in patients, especially those with bloodstream infections (BSIs), consistently resulting in fatal outcomes.^7^ Further, increasing rates of hypervirulent *K. pneumoniae* infections have been reported worldwide.^8^ Therefore, knowledge of risk factors associated with the development of CPKP infections and mortality may be helpful in controlling the spread of CPKP, treatment expenses, and survival rate.

Although several studies have evaluated risk factors for CPKP infections, the results have been inconsistent.^9–11^ Many studies were retrospective analyses, but few studies have investigated the epidemiology of CPKP. The aim of this study was to describe the microbiological and clinical characteristics and the economic burden of CPKP infections in sterile and relatively sterile body sites, such as the biliary tract, urinary tract, pleural areas, abdominal cavity, and blood. This study also assessed the risk factors associated with carbapenem resistance and patient mortality.

## Methods

### Study design and setting

This matched retrospective cohort study assessed the incidence, risk factors, antibiotic resistance, and medical costs associated with the acquisition of health care-associated *K. pneumoniae* infections (excluding sputum specimens, tracheal secretions, and broncho-alveolar lavage fluid) in patients admitted to the First Affiliated Hospital of Zhejiang University in 2014. Patients infected with CPKP were compared with patients infected with carbapenem-susceptible *K. pneumoniae* (CSKP) to assess risk factors for antibiotic resistance and patient mortality. The two groups were matched by age, sex, and specimen source in a 1:2 ratio.

Subjects with CPKP or CSKP isolated from multiple sites, or on multiple dates, were counted only once, with the data from the first infection included in the study. Patients below age 16 years were excluded. Health care-acquired CPKP or CSKP infection was defined as isolation 48 hours after admission to the hospital. CPKP was defined according to the updated 2015 Centers for Disease Control and Prevention guidelines.^12^ Patients with CPKP or CSKP colonization and those with community-acquired infection were excluded.

### Bacterial strains

Pathogens were isolated by standard microbiological methods and identified by using VITEK 2 automated instrument for ID/AST testing (Bio-Mérieux, France). All pure cultures were frozen at −80°C and shipped to a central laboratory for definitive identification and further analysis. Species identification was confirmed by matrix-assisted laser desorption ionization time of flight mass spectrometry (MALDI-TOF; VITEK MS, bioMérieux, Nürtingen, Germany). Carbapenem-resistant *K. pneumoniae* (CRKP) strains were pre-selected based on VITEK2 susceptibility results that were compatible with minimal inhibitory concentrations (MICs) of imipenem ≥4 mg/L, meropenem ≥4 mg/L, or ertapenem ≥2 mg/L. Carbapenemase-producing isolates were identified by using a modified Hodge test (MHT), according to Clinical and Laboratory Standards Institute (CLSI) guidelines,^13^ with *Escherichia coli* ATCC 25922 and *K. pneumoniae* ATCC BAA-1705 and BAA-1706 used as reference strains. Isolates with positive MHT results were included in this study. Carbapenemase genes were routinely amplified by PCR.^14^ Multi-locus sequence typing (MLST) was performed on all CPKP isolates by using the scheme of the Institute Pasteur.^15^

### Antibiotic susceptibility testing

Susceptibilities of CPKP strains to antimicrobial agents were determined by Mueller-Hinton agar dilution, as described by CLSI guidelines.^13^ The MIC of tigecycline was measured by broth microdilution as recommended. The 23 antimicrobial agents tested included amikacin, aztreonam, cefazolin, cefepime, ceftazidime, ceftriaxone, ciprofloxacin, gentamicin, imipenem, levofloxacin, piperacillin-tazobactam, trimethoprim-sulfamethoxazole, meropenem, fosfomycin, amoxicillin, amoxicillin-clavulanic acid, cefuroxime, cefoxitin, cefoperazone-sulbactam, polymyxin B, moxalactam, ertapenem, and colistin. The antimicrobial susceptibilities of CSKP were also tested by VITEK 2. EUCAST breakpoint recommendations were chosen for fosfomycin, tigecycline, and colistin.^16^ The results for other antibiotics were interpreted according to CLSI criteria.^13^ Multidrug-resistant (MDR) and extensively drug-resistant (XDR) strains were defined as previously described.^17^

### Characterization of the hypervirulent phenotype and virulence genes

Hypervirulent phenotypes were identified by using the string test.^8^ CPKP strains positive on string tests were designated as hypervirulent variants of CPKP (hvCPKP).

K1^18^ and K2^19^ capsular serotypes and virulence-associated genes, including *rmpA*,^20^ *mrkD*,^21^ *entB*,^21^ *ybtS*,^21^ *fimH*,^22^ kpn,^21^ *iutA*,^21^ *alls*^21^ *wcaG*,^23^ *kfuB*,^21^ and *magA*,^24^ were identified by PCR amplification and DNA sequencing.

### Medical records

The medical records of patients who presented with laboratory-confirmed CPKP and CSKP were reviewed by two researchers. Data recorded included demographic characteristics, clinical characteristics (underlying diseases, comorbidities, invasive procedures, and surgical procedures), results of laboratory examinations, treatment history, antimicrobials, hospitalization, and clinical outcomes. Drug costs were also recorded. The Acute Physiology and Chronic Health Evaluation (APACHE) II score and Pitt bacteremia score were calculated to assess the severity of illness.^25,26^

Steroid therapy was defined as >20 mg/day prednisone or its equivalent administered for ≥7 days. Antimicrobial drug exposure was defined as the treatment with any antibiotic for >72 hours at any point 2 weeks before diagnosis. Mortality was defined as death during hospitalization or up to 28 days after discharge, as confirmed by telephone follow-up. Overall mortality included all causes of death during hospitalization.

### Statistical analysis

Continuous variables were expressed as mean ± standard deviation and compared by *t* tests. Categorical variables were expressed as numbers and percentages and compared by chi-square tests. For multivariate analysis, binary logistic regression was used to identify risk factors. A two-tailed *p*-value <0.05 was considered statistically significant. All statistical analyses were performed by using SPSS 23.0 for Windows (SPSS, Inc., Chicago, IL).

## Results

### Incidence of CPKP infection

**FIG. 1.**
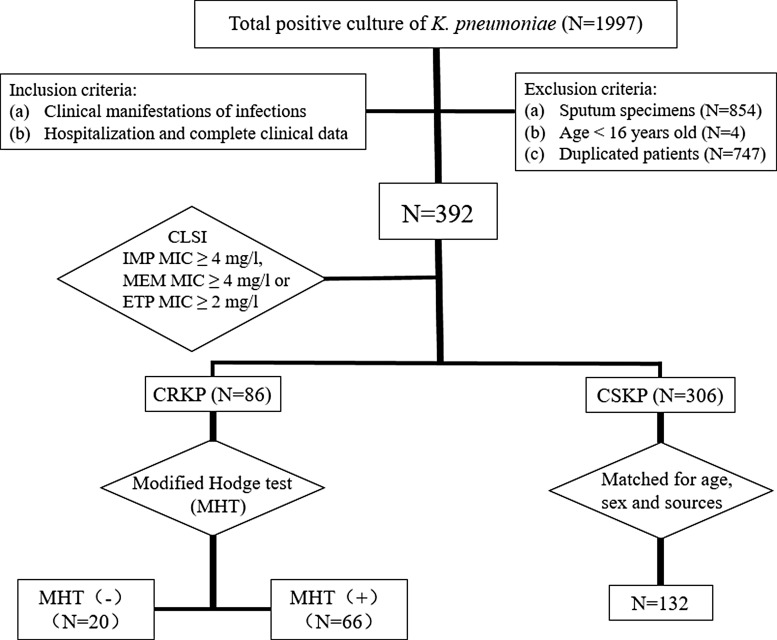
Flowchart of the patient selection process. IMP, imipenem; ETP, ertapenem; CPKP, carbapenemase-producing *Klebsiella pneumoniae*; CSKP, carbapenem-susceptible *K. pneumoniae*.

The overall incidence of CPKP, excluding sputum specimens, tracheal secretions, and broncho-alveolar lavage fluid, was 21.9% (86/392) in 2014. Sixty-six carbapenem-resistant isolates were positive for production of carbapenemases. These patients were matched 1:2 with 132 CSKP infected patients (Fig. 1).

### Molecular epidemiology characteristics of CPKP strains

**FIG. 2.**
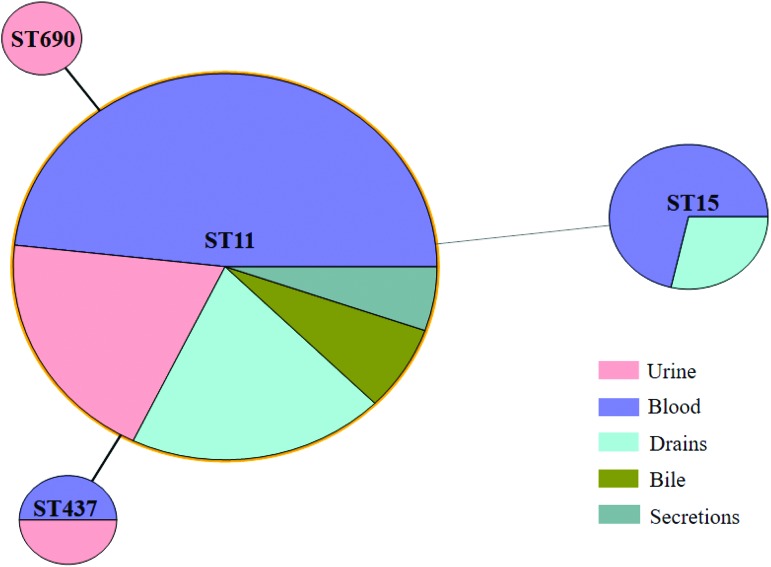
Minimum spanning tree of CPKP. *Solid line* indicates one allele difference, and *dashed line* indicates differences in two alleles. Color images are available online.

The 66 CPKP isolates included four different STs. The most prevalent was ST11 (56 isolates, 84.8%), followed by ST15 (7 isolates, 10.6%), ST437 (2 isolates, 3.1%), and ST690 (1 isolate, 1.5%) (Fig. 2). All CPKP strains examined in this study were positive for *bla*_kpc-2_.

Of these 66 CPKP strains, 3 (4.5%) had capsular genotype K1 and 23 (34.8%) had capsular genotype K2. The rates of *mrkD*, *entB*, *ybtS*, *fimH*, *kpn*, *iutA*, *rmpA*, and *kfu* positivity were 98.5%, 90.9%, 89.4%, 87.9%, 84.8%, 48.5%, 10.6%, and 9.1%, respectively. Of the 66 CPKP isolates, 15 (22.7%) were positive on string tests. Of these 15 hvCPKP strains, 13 (86.7%) were ST11, with 1 each being ST143 and ST314. The coexistence of *entB*, *ybtS*, and *iutA* was significantly higher in hvCPKP than in non-hvCPKP strains (*p* = 0.022) (Table 1).

**Table 1.** Rates of Virulence-Associated Phenotypes and Genes Among 66 Carbapenemase-Producing *Klebsiella pneumoniae* Isolates

	*hvCPKP* n *(%)*	*non-hvCPKP* n *(%)*	p
Phenotype	15 (22.7)	51 (77.3)	—
K1	0 (0)	3 (5.9)	0.340
K2	1 (6.7)	22 (43.1)	0.010
*rmpA*	3 (20)	4 (7.8)	0.182
*fimH*	14 (93.3)	44 (86.3)	0.465
*mrkD*	15 (100)	50 (98)	0.588
*kpn*	13 (86.7)	43 (84.3)	0.825
*entB*	14 (93.3)	46 (90.2)	0.712
*ybtS*	13 (86.7)	46 (90.2)	0.669
*iutA*	10 (66.7)	22 (43.1)	0.112
*kfu*	0 (0)	6 (11.8)	0.167
*magA*	0 (0)	0 (0)	—
*alls*	0 (0)	0 (0)	—
*wcaG*	0 (0)	0 (0)	—
*entB+ybtS+iutA*	10 (66.7)	17 (33.3)	0.022

hvCPKP, hypervirulent variant of carbapenemase-producing *K. pneumoniae*; non-hvCPKP, non-hypervirulent variant of carbapenemase-producing *K. pneumonia*e.

### Antibiotic susceptibility test

Antimicrobial susceptibilities of all isolates are presented in Tables 2 and 3. Sixty-six strains were MDR CPKP strains, and 31 were XDR CPKP strains. All CPKP strains were resistant to ertapenem, whereas 4.5% and 7.6% were susceptible to imipenem and meropenem, respectively. Only one isolate was resistant to colistin, whereas two were resistant to polymyxin B. However, 40.9% and 37.9% of CPKP isolates were susceptible to fosfomycin and amikacin, respectively (Table 3), whereas 27.3% were resistant to tigecycline.

**Table 2.** Minimum Inhibitory Concentrations of 18 Antimicrobial Agents Against 132 Carbapenem-Susceptible *K. pneumoniae* Isolates

*Drugs*	*MIC range (mg/L)*	*MIC50 (mg/L)*	*MIC90 (mg/L)*	*S* n *(%)*	*R* n *(%)*
AMK	≤2 to >32	≤2	≤2	128 (97)	4 (3)
AMP	≤2 to ≥32	≥32	≥32	0 (100)	132 (100)
ATM	≤1 to ≥32	≤1	≥32	103 (78)	28 (21.2)
CAZ	≤1 to >32	≤1	16	101 (76.5)	23 (17.4)
CIP	≤0.25 to >4	≤0.25	>4	99 (75)	28 (21.2)
CRO	≤1 to >32	≤1	>32	96 (72.7)	35 (26.5)
CTT	≤4 to >64	4	4	131 (99.2)	1 (0.8)
CZO	≤2 to >32	4	>32	61 (46.2)	44 (33.3)
ETP	≤0.25 to 1	≤0.25	0.5	129 (97.7)	0 (0)
FEP	≤1 to >32	≤1	32	115 (87.1)	13 (9.8)
GEN	≤1 to ≥16	≤1	≥16	103 (78)	29 (22)
IMP	≤0.5 to 2	≤0.5	≤0.5	120 (90.9)	0 (0)
LVX	≤0.25 to >8	≤0.25	>8	103 (78)	23 (17.4)
NIT	≤16 to >256	64	256	19 (14.4)	44 (33.3)
SAM	≤2/1 to ≥32/16	8	≥32/16	73 (55.3)	49 (37.1)
SXT	≤1/19 to >8/152	≤1/19	>8/152	98 (74.2)	34 (25.8)
TOB	≤1 to ≥16	≤1	8	103 (78)	10 (7.5)
TZP	≤4/4 to ≥128/4	≤4/4	16/4	120 (90.9)	7 (5.3)

AMK, amikacin; AMP, ampicillin; ATM, aztreonam; CAZ, ceftazidime; CIP, ciprofloxacin; CRO, ceftriaxone; CTT, cefotetan; CZO, cefazolin; ETP, ertapenem; FEP, cefepime; GEN, gentamicin; IMP, imipenem; LVX, levofloxacin; MIC, minimal inhibitory concentration; NIT, nitrofurantoin; R, resistant; S, susceptible; SAM, ampicillin-sulbactam; SXT, trimethoprim-sulfamethoxazole; TOB, tobramycin; TZP, piperacillin-tazobactam.

**Table 3.** Minimum Inhibitory Concentrations of 24 Antimicrobial Agents Against 66 Carbapenemase-Producing *K. pneumoniae* Islolates

*Drugs*	*MIC range (mg/L)*	*MIC50 (mg/L)*	*MIC90 (mg/L)*	*S* n *(%)*	*R* n *(%)*
AMC	≥128/64	128/64	>128/64	0 (0)	66 (100)
AMK	1 to >2,048	>2,048	>2,048	25 (37.9)	41 (62.1)
AMX	>256	>256	>256	0 (0)	66 (100)
ATM	128 to >128	>128	>128	0 (0)	66 (100)
CAZ	16 to >128	64	>128	0 (0)	66 (100)
CIP	2 to >128	128	>128	0 (0)	65 (98.5)
COL	0.15 to >32	0.25	0.5	65 (98.5)	1 (1.5)
CRO	64 to >128	>128	>128	0 (0)	66 (100)
CSL (1:1)	1/0.5 to >128/64	>128/64	>128/64	1 (1.5)	64 (97)
CXM	>128	>128	>128	0 (0)	66 (100)
CZO	>128	>128	>128	0 (0)	66 (100)
ETP	2 to >1,024	128	512	0 (0)	66 (100)
FEP	8 to >128	128	>128	2 (3)	61 (92.4)
FM	2 to >2,048	512	>2,048	27 (40.9)	39 (59.1)
FOX	8 to >128	>128	>128	3 (4.5)	63 (95.5)
GEN	0.25 to >128	>128	>128	13 (19.7)	53 (80.3)
IMP	0.5 to 1,024	16	64	3 (4.5)	60 (90.9)
LVX	8 to >128	32	128	0 (0)	66 (100)
MEM	0.5 to >32	>32	>32	5 (7.6)	61 (92.4)
MOX	4 to >128	>128	>128	1 (1.5)	10 (15.2)
PB	0.5 to >32	1	1	64 (97)	2 (3)
SXT	0.03/0.57 to >8/152	>8/152	>8/152	21 (31.8)	45 (68.2)
TGC	1 to 16	2	4	26 (39.4)	18 (27.3)
TZP	2/4– >128/4	>128/4	>128/4	1 (1.5)	65 (98.5)

AMC, amoxicillin-clavulanic acid; AMX, amoxicillin; COL, colistin; CSL, cefoperazone-sulbactam; CXM, cefuroxime; FM, fosfomycin; FOX, cefoxitin; MEM, meropenem; MOX, moxalactam; PB, polymyxin B; TGC, tigecycline.

### Patient demographics and specimen types

**FIG. 3.**
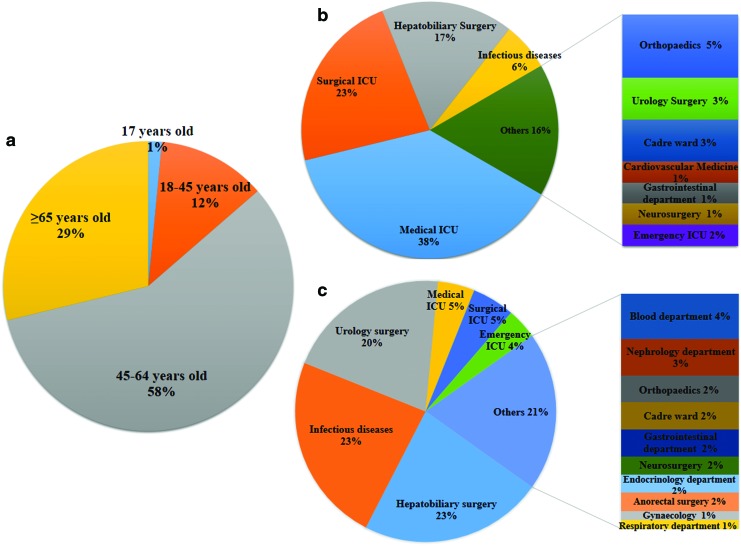
Patient demographics. **(a)** Ages of patients with CPKP infections; **(b, c)** Wards from which **(b)** CPKP and **(c)** CSKP strains were isolated. Color images are available online.

The demographics and clinical characteristics of the 198 patients infected with *K. pneumoniae* are shown in Supplementary Table S1. Of the 66 CPKP isolates, 28 were recovered from blood, followed by drains (*n* = 17), urine (*n* = 16), bile (*n* = 3), and secretions (*n* = 2). Forty-five (68.2%) isolates were recovered from men, and 21 (31.8%) were obtained from women. When sorted by age, 1%, 12%, 58%, and 29% of strains were recovered from patients aged 17, 18–45, 46–64, and ≥65 years, respectively (Fig. 3a).

The highest incidence of CPKP infections was observed in intensive care units (ICUs), with 38%, 23%, and 2% recovered from patients in the medical, surgical, and emergency ICUs, respectively. In addition, 17% and 6% of strains were recovered from patients in the hepatobiliary surgery and infectious diseases departments, respectively. By contrast, 23% of CSKP strains each were recovered from patients in the hepatobiliary surgery and infectious diseases departments, and 20% were obtained from patients in the urology surgery department (Fig. 3b, c).

Overall, the median duration of hospitalization was significantly longer for CPKP (46 days; range 7–218 days) than for CSKP (23 days; range 1–101 days) infected patients (*p* < 0.001). The crude in-hospital mortality was also significantly higher for patients infected with CPKP than for those infected with CSKP (57.6% [38/66] vs. 18.2% [24/132], *p* < 0.001) (Supplementary Table S1).

### Risk factors associated with the development of CPKP

Univariate analysis showed that, compared with patients with CSKP, patients with CPKP were more likely to have coronary heart disease, cerebral infarction, renal insufficiency, or an organ transplant, or to have undergone a nonsurgical invasive procedure or hemodialysis. Patients with CPKP also had lower albumin concentrations and higher APACHE II and Pitt bacteremia scores, and they were more likely to have received corticosteroid therapy or to have been exposed to antimicrobial therapy in the previous 14 days (Supplementary Table S2). Multivariate logistic regression analysis (Table 4) showed that risk factors for CPKP included Pitt bacteremia score >4 (odds ratio [OR] = 7.677, 95% confidence interval [CI] = 2.960–19.915, *p* < 0.001), prior stomach tube placement (OR = 5.350, 95% CI = 2.225–12.867, *p* < 0.001), continuous renal replacement therapy (CRRT) (OR = 3.565, 95% CI = 1.286–9.881, *p* = 0.015), and previous carbapenem exposure (OR = 8.096, 95% CI = 2.404–27.262, *p* = 0.001).

**Table 4.** Logistic Regression Model of Risk Factors for Development of Carbapenemase-Producing K. pneumoniae

	*Univariate analysis*			*Multivariable analysis*			
				*95% CI for EXP(B)*			
	*CPKP (*n* = 66)* n *(%)*	*CSKP (*n* = 132)* n *(%)*	p	*Sig.*	*Exp(B)*	*Lower*	*Upper*
Pitt bacteremia score >4	31 (47)	8 (6.1)	<0.001	<0.001	7.677	2.960	19.915
Prior stomach tube placement	56 (84.8)	49 (37.1)	<0.001	<0.001	5.350	2.225	12.867
CRRT	22 (33.3)	9 (6.8)	<0.001	0.015	3.565	1.286	9.881
Previous carbapenem exposure	17 (25.8)	6 (4.5)	<0.001	0.001	8.096	2.404	27.262

CI, confidence interval; CSKP, carbapenem-susceptible *K. pneumoniae*; CRRT, continuous renal replacement therapy.

*K. pneumoniae*, identified as the first pathogen, was present in 18.2% (12/66) of CPKP patients and in 68.2% (90/132) of CSKP patients. The rates of concomitant infection with other bacteria were 89.4% (59/66) in CPKP patients and 48.5% (64/132) in CSKP patients.

### Risk factors for crude 28-day mortality in patients

Of the 198 patients, 27 died within 28 days, 12 with CPKP and 15 with CSKP, and 171 survived (Supplementary Table S3). Multivariate logistic regression analysis showed that risk factors for death included Pitt bacteremia score >4 (OR = 3.802, 95% CI = 1.470–9.840, *p* = 0.006) and neutrophil percentage (OR = 1.069, 95% CI = 1.012–1.128, *p* = 0.016). Higher serum albumin concentration (OR = 0.306, 95% CI = 0.137–0.685, *p* = 0.004) and treatment with cephalosporins after diagnosis (OR = 0.207, 95% CI = 0.043–0.998, *p* = 0.049) were associated with a lower risk of death.

### Risk factors for crude in-hospital mortality

Logistic regression analysis showed that risk factors for all-cause mortality included high APACHE II score (OR = 1.120, 95% CI = 1.051–1.194, *p* < 0.001), CRRT (OR = 3.091, 95% CI = 1.137–8.404, *p* = 0.027), treatment with carbapenems after diagnosis (OR = 3.079, 95% CI = 1.203–7.883, *p* = 0.019), and bacteremia (OR = 2.824, 95% CI = 1.295–6.158, *p* = 0.009) (Table 5 and Supplementary Table S4).

**Table 5.** Logistic Regression Model of Risk Factors for Crude In-Hospital Mortality

	*Univariate analysis*			*Multivariable analysis*			
				*95% CI for EXP(B)*			
	*Death (*n* = 62)* n *(%)*	*Survivors (*n* = 136)* n *(%)*	p	*Sig.*	*Exp(B)*	*Lower*	*Upper*
APACHE II score	14.1 ± 5.9	8.6 ± 5.3	<0.001	<0.001	1.120	1.051	1.194
CRRT	22 (35.5)	9 (6.6)	<0.001	0.027	3.091	1.137	8.404
Carbapenem after diagnosis	55 (88.7)	72 (52.9)	<0.001	0.019	3.079	1.203	7.883
Bacteremia	36 (58.1)	24 (17.6)	<0.001	0.009	2.824	1.295	6.158

APACHE II score, Acute Physiology and Chronic Health Evaluation II score.

The most commonly used treatment agents were β-lactam and/or β-lactamase inhibitor (*n* = 134, 67.7%), followed by carbapenems (*n* = 127, 64.1%), cephalosporins (*n* = 49, 24.7%), fluoroquinolones (*n* = 44, 22.2%), and tigecycline (*n* = 34, 18.7%). Treatment with carbapenems, however, was an independent risk factor for increased crude in-hospital mortality in patients with *K. pneumoniae* infections. In addition, among CPKP patients, there was no significant difference in crude in-hospital mortality between patients infected with hvCPKP and non-hvCPKP strains (60% vs. 56.9%, *p* = 0.829).

### Antibiotic costs

A comparison of the costs of anti-infective drugs used by patients in the CPKP and CSKP groups showed that median costs were significantly higher for the CPKP than for the CSKP group (3903.7€ vs. 786.6€, *p* < 0.001) (Supplementary Table S1).

## Discussion

Due to the extensive use of antibiotics, the rates of CPKP infection, even of MDR and XDR strains, are increasing.^4^ This analysis of KPC2-producing *K. pneumoniae* showed that MDR CPKP was spread by clonal strains, resulting in higher antibiotic costs. In addition, Pitt bacteremia score >4, prior stomach tube placement, CRRT, and previous exposure to carbapenems were risk factors for the development of CPKP infections. Further, bacteremia, disease severity, CRRT, and treatment with carbapenems after diagnosis were identified as risk factors for increased mortality rates.

The highest incidence of CPKP infections was observed in ICUs, with 69.7% of CPKP patients admitted to the ICU before infection. Usually, ICU patients are in critical condition with lower immunity and higher APACHE II scores. These patients receive extensive antibiotic therapy and undergo invasive procedures, making them more vulnerable to CPKP infections.^27^ These findings confirm the results of previous studies, showing that the severity of disease is an important risk factor for CPKP infections.

Interestingly, we found that prior stomach tube placement was independently associated with CPKP infections. *K. pneumoniae*, including CPKP strains, colonizes the intestinal tract. Changes in diet and use of antibiotics can cause imbalances in intestinal flora and reduce bacterial translocation, resulting in damage to intestinal barrier function.^28^ Asymptomatic colonization of the gastrointestinal tract by carbapenemase-producing Enterobacteriaceae resulted in an important reservoir for transmission that may precede infection.^29^ In addition, previous BSIs by other pathogens were associated with an increased risk of CRKP BSI, independent of other factors in colonized patients with prolonged hospital exposure.^30^ Long-term colonization of patients with *bla*_KPC_-positive *K. pneumoniae* creates new opportunities for horizontal gene transfer of plasmids encoding antibiotic resistance genes and poses complications for the delivery of health care.^31^

Besides observing clinical characteristics, we also performed molecular analysis of isolated CPKP strains to analyze the mechanisms of antimicrobial resistance, and to rule out the possibility of an outbreak during the study period. The most abundant MLST type among CPKP strains was ST11 (84.8%), followed by ST15 (10.6%), ST437 (3.1%), and ST690 (1.5%). In Asia, the dominant clone of KPC-positive *K. pneumoniae* is ST11, which is different from the United States, in which ST258 is the dominant clone.^32^ Further, ST11 (13/15, 86.7%) was dominant among hvCPKP strains. The prevalence of hypervirulent *K. pneumoniae* in previous Chinese studies was significantly higher than that in studies performed in Alberta, Canada, and Spain.^33^ The emergence of ST11 hypervirulent CRKP strains was shown to be due to the acquisition of a 170 kbp pLVPK-like virulence plasmid, causing fatal ventilator-associated pneumonia in hospitalized patients.^7^ We also observed increases in hvCPKP isolates in clinical settings, suggesting the need for immediate responses to MDR hypervirulent *K. pneumoniae* infections. However, crude in-hospital mortality rates were similar in patients infected with hvCPKP and non-hvCPKP strains (60% vs. 56.9%, *p* = 0.829). Similarly, the 30-day mortality rate was lower in hypervirulent *K. pneumoniae* infected patients than in classic *K. pneumoniae* infected patients (4.5% vs. 16.7%).^34^ Due to the limited sample size in these studies, large-scale, multicenter clinical control studies are needed to assess the influence of hvCPKP on mortality.

Further exploration of the high mortality rate showed that the 28-day death rate associated with CPKP was 44.4%, not significantly higher than that associated with CSKP (31.6%) (*p* = 0.188). However, the in-hospital death rate was significantly higher in the CPKP than in the CSKP group (61.3% vs. 20.6%) (*p* < 0.001). One possible explanation is that the median duration of hospitalization was longer in CPKP than in CSKP infected patients (46 days vs. 23 days). APACHE II score, CRRT, and bacteremia were risk factors for crude in-hospital mortality in discharged patients, indicating that disease severity was the principal cause of death. Multivariate analysis showed that treatment with carbapenems after diagnosis was an independent risk factor for death in discharged patients, whereas it was protective for crude 28-day mortality. Early carbapenem treatment should be considered for patients at high risk of invasive ESBL infections,^35^ whereas carbapenem treatment of Enterobacteriaceae with MICs ≥8 mg/L is not recommended.^36,37^

At present, there are no clear guidelines for drug selection in treating CPKP infections. Treatment with combinations of antibiotics is associated with significantly better survival than monotherapy (69.4% vs. 42.6%, *p* < 0.001).^38^ A previous study showed that the 28-day mortality rate was 13.3% in the combination therapy group compared with 57.8% in the monotherapy group.^39^ Combination therapy with at least two drugs displaying *in vitro* activity against the isolate was associated with lower mortality rates, in particular in patients with BSIs, lung infections or high APACHE II scores, and/or septic shock at infection onset.^40^ Additional clinical trials of combination therapy are urgently needed to guide therapeutic options for deep-seated CPKP infections.

This study had several limitations, including its retrospective design and the relatively small number of patients infected with CPKP. However, this is a real-life clinical study providing useful suggestions about the management of difficult-to-treat and poorly studied infections caused by CPKP.

In conclusion, Pitt bacteremia score >4, prior stomach tube placement, CRRT, and previous exposure to carbapenems were significant risk factors for the development of CPKP. In addition, CPKP infections were associated with a high financial burden. Critical illness significantly increased patient mortality rates.

## Ethical Approval

This study was approved by the Ethics Committee of all participating institutions (Reference No.: 2017646). All subjects provided written informed consent in accordance with the Declaration of Helsinki.

## Supplementary Material

Supplemental data

Supplemental data

Supplemental data

Supplemental data
