# Supplementary material for: Infections Caused by Carbapenemase-Producing Klebsiella pneumoniae: Microbiological Characteristics and Risk Factors
Source: Microb Drug Resist. 2019 Mar 8;25(2):287–96. doi: 10.1089/mdr.2018.0339 (PMC6441289; doi:10.1089/mdr.2018.0339)
Supplement: Supplemental data [file Supp_Table1.pdf]

Supplementary Data

Supplementary Table S1. Clinical and Demographic Characteristics of Patients with *Klebsiella pneumoniae* Infections

	CPKP (n = *66*)	*CSKP* (n = *132*)
Age (years)	58.8 ± 15.9	57.4 ± 14.7
Gender, male, *n* (%)	45 (68.2)	90 (68.2)
APACHE II score	13 (8–18)	8 (5–12)
Pitt bacteremia score >4, *n* (%)	31 (47)	8 (6.1)
ICU stay, *n* (%)	46 (69.7)	31 (23.5)
Hospital day (days)	46 (26–81)	23 (12–37)
Death, *n* (%)	38 (57.6)	24 (18.2)
Costs (RMB)	30398.7 (13842.6–62854.8)	6125.3 (2365.4–15850.8)
K. pneumoniae identified as the first pathogens, *n* (%)	12 (18.2)	90 (68.2)
Single pathogen, *n* (%)	7 (10.6)	68 (51.5)
Concomitant G– infection, *n* (%)	48 (72.7)	41 (31.1)
Concomitant G+ infection, *n* (%)	36 (54.5)	30 (22.7)
Concomitant fungus infection, *n* (%)	29 (43.9)	21 (15.9)
Metastatic infection, *n* (%)	21 (31.8)	18 (13.6)
Bacteremia, *n* (%)	44 (66.7)	16 (12.1)
Admission due to bacteremia, *n* (%)	9 (13.6)	9 (6.8)
Admission due to sepsis, *n* (%)	15 (22.7)	8 (6.1)
Invasive procedure or devices		
Surgery, *n* (%)	34 (51.5)	74 (56.1)
Urinary catheterization, *n* (%)	61 (92.4)	81 (61.4)
Venous catheterization, *n* (%)	65 (98.5)	130 (98.5)
Arterial catheterization, *n* (%)	50 (75.8)	52 (39.4)
Stomach tube, *n* (%)	56 (84.8)	49 (37.1)
Mechanical ventilation, *n* (%)	46 (69.7)	31 (23.5)
Tracheotomy, *n* (%)	29 (43.9)	11 (8.3)
Continuous renal replacement therapy, *n* (%)	22 (33.3)	9 (6.8)
Hemodialysis, *n* (%)	23 (34.8)	13 (9.8)
Bronchofibroscope use, *n* (%)	1 (1.5)	3 (2.3)
Wound drainage tube use, *n* (%)	44 (66.7)	88 (66.7)
Prior chemotherapy or radiotherapy, *n* (%)	5 (7.6)	9 (6.8)
Prior corticosteroid therapy, *n* (%)	27 (40.9)	23 (17.4)
Prior immunosuppressant use, *n* (%)	9 (13.6)	9 (6.8)
Prior antimicrobial therapy in the previous 14 days		
β-lactam and/or β-lactamase inhibitor, *n* (%)	18 (27.3)	13 (9.8)
Cephalosporins, *n* (%)	11 (16.7)	7 (5.3)
Carbapenems, *n* (%)	17 (25.8)	6 (4.5)
Fluoroquinolone, *n* (%)	10 (15.2)	7 (5.3)
Aminoglycoside, *n* (%)	2 (3.0)	1 (0.7)
Vancomycin, *n* (%)	7 (10.6)	0
Tigecycline, *n* (%)	4 (6.1)	1 (0.8)
Teicoplanin, *n* (%)	3 (4.5)	3 (2.3)
Ornidazole, *n* (%)	1 (1.5)	2 (1.5)
Linezolid, *n* (%)	0	1 (0.8)
Pre-existing medical conditions		
Diabetes, *n* (%)	9 (13.6)	27 (20.5)
Hepatitis, *n* (%)	11 (16.7)	22 (16.7)
Tumor, *n* (%)	17 (25.8)	42 (31.8)
Hypertension, *n* (%)	19 (28.8)	38 (28.8)
Coronary heart disease, *n* (%)	5 (7.6)	0
Cerebral infarction, *n* (%)	3 (4.5)	0
Renal insufficiency, *n* (%)	3 (4.5)	0
Trauma, *n* (%)	1 (1.5)	1 (0.8)
Organ transplant, *n* (%)	2 (3.0)	0
Antimicrobial therapy after diagnosis		
β-lactam and/or β-lactamase inhibitor, *n* (%)	45 (68.2)	89 (67.4)

(*continued*)

Supplementary Table S1. (Continued)

	CPKP (n=*66)*	*CSKP* (n =*132)*
Cephalosporins, *n* (%)	13 (19.7)	36 (27.3)
Carbapenems, *n* (%)	54 (81.8)	73 (55.3)
Fluoroquinolone, *n* (%)	15 (22.7)	29 (22)
Aminoglycoside, *n* (%)	12 (18.2)	4 (3.0)
Vancomycin, *n* (%)	17 (25.8)	12 (9.1)
Tigecycline, *n* (%)	26 (39.4)	8 (6.1)
Teicoplanin, *n* (%)	9 (13.6)	22 (16.7)
Ornidazole, *n* (%)	1 (1.5)	14 (10.6)
Linezolid, *n* (%)	4 (6.1)	4 (3.0)
Fosfomycin, *n* (%)	4 (6.1)	7 (5.3)
Daptomycin, *n* (%)	3 (4.5)	1 (0.8)
Combination therapy, *n* (%)	50 (75.8)	51 (38.6)
Laboratory examination		
White blood cell (10E9/L)	12.0 ± 9.7	9.2 ± 5.3
Neutrophil percentage (%)	86.8 (72.6–93.2)	78.9 (65.7–86.8)
Hemoglobin (g/L)	96.8 ± 27.5	120.5 ± 100.8
Platelet (10E9/L)	185.7 ± 133.9	194.1 ± 125.2
Hypersensitivity C reactive protein (mg/L)	100.5 ± 82.1	72.2 ± 58.4
Albumin (g/L)	31.8 ± 5	33.7 ± 7.3
Alanine transaminase (U/L)	25.5 (12–72)	12.00
Aspartate aminotransferase (U/L)	28.5 (12–75.8)	21.00
Cholinesterase (U/L)	3496.8 ± 1853.2	4648.5 ± 2368.3
Total bilirubin (μmol/L)	15.5 (10–47)	15.5 (10–34)
Serum creatinine (μmol/L)	65 (47.8–113.8)	67.5 (52.3–92.8)
INR	1.1 (1.0–1.2)	1.0 (0.9–1.2)

APACHE II score, Acute Physiology and Chronic Health Evaluation II score; CPKP, carbapenemase-producing *K. pneumoniae*; CSKP, carbapenem-susceptible *K. pneumoniae*; ICU, intensive care unit; INR, international normalized ratio; RMB, Renminbi, Chinese yuan.
