# Supplementary material for: Infections Caused by Carbapenemase-Producing Klebsiella pneumoniae: Microbiological Characteristics and Risk Factors
Source: Microb Drug Resist. 2019 Mar 8;25(2):287–96. doi: 10.1089/mdr.2018.0339 (PMC6441289; doi:10.1089/mdr.2018.0339)
Supplement: Supplemental data [file Supp_Table2.pdf]

Supplementary Table S2. Risk Factors for Patients with Carbapenemase-Producing *Klebsiella pneumoniae* at Univariate Analysis

	CPKP (n=*66)*	*CSKP* (n=*132)*	p
Age (years)	58.8 ± 15.9	57.4 ± 14.7	0.545
Gender, male, *n* (%)	45 (68.2)	90 (68.2)	0.831
APACHE II score	13.2 ± 6.2	8.9 ± 5.5	<0.001
Pitt bacteremia score >4, *n* (%)	31 (47)	8 (6.1)	<0.001
ICU stay, *n* (%)	46 (69.7)	31 (23.5)	<0.001
Invasive procedure or devices			
Surgery, *n* (%)	34 (51.5)	74 (56.1)	0.545
Urinary catheterization, *n* (%)	61 (92.4)	81 (61.4)	<0.001
Venous catheterization, *n* (%)	65 (98.5)	130 (98.5)	1
Arterial catheterization, *n* (%)	50 (75.8)	52 (39.4)	<0.001
Stomach tube, *n* (%)	56 (84.8)	49 (37.1)	<0.001
Mechanical ventilation, *n* (%)	46 (69.7)	31 (23.5)	<0.001
Tracheotomy, *n* (%)	29 (43.9)	11 (8.3)	<0.001
Continuous renal replacement therapy, *n* (%)	22 (33.3)	9 (6.8)	<0.001
Hemodialysis, *n* (%)	23 (34.8)	13 (9.8)	<0.001
Bronchofibroscope use, *n* (%)	1 (1.5)	3 (2.3)	0.721
Wound drainage tube use, *n* (%)	44 (66.7)	88 (66.7)	1
Prior chemotherapy or radiotherapy, *n* (%)	5 (7.6)	9 (6.8)	0.845
Prior corticosteroid therapy, *n* (%)	27 (40.9)	23 (17.4)	<0.001
Prior immunosuppressant use, *n* (%)	9 (13.6)	9 (6.8)	0.116
Prior antimicrobial therapy in the previous 14 days			
β-lactam and/or β-lactamase inhibitor, *n* (%)	18 (27.3)	13 (9.8)	0.001
Cephalosporins, *n* (%)	11 (16.7)	7 (5.3)	0.009
Carbapenems, *n* (%)	17 (25.8)	6 (4.5)	<0.001
Fluoroquinolone, *n* (%)	10 (15.2)	7 (5.3)	0.02
Aminoglycoside, *n* (%)	2 (3.0)	1 (0.7)	0.217
Vancomycin, *n* (%)	7 (10.6)	0	<0.001
Tigecycline, *n* (%)	4 (6.1)	1 (0.8)	0.025
Teicoplanin, *n* (%)	3 (4.5)	3 (2.3)	0.379
Ornidazole, *n* (%)	1 (1.5)	2 (1.5)	1
Linezolid, *n* (%)	0	1 (0.8)	0.478
Pre-existing medical conditions			
Diabetes, *n* (%)	9 (13.6)	27 (20.5)	0.241
Hepatitis, *n* (%)	11 (16.7)	22 (16.7)	1
Tumor, *n* (%)	17 (25.8)	42 (31.8)	0.379
Hypertension, *n* (%)	19 (28.8)	38 (28.8)	1
Coronary heart disease, *n* (%)	5 (7.6)	0	0.001
Cerebral infarction, *n* (%)	3 (4.5)	0	0.014
Renal insufficiency, *n* (%)	3 (4.5)	0	0.014
Trauma, *n* (%)	1 (1.5)	1 (0.8)	0.615
Organ transplant, *n* (%)	2 (3.0)	0	0.044
Laboratory examination			
White blood cell (10E9/L)	12.0 ± 9.7	9.2 ± 5.3	0.029
Neutrophil percentage (%)	86.8 (72.6–93.2)	78.9 (65.7–86.8)	0.005
Hemoglobin (g/L)	96.8 ± 27.5	120.5 ± 100.8	0.062
Platelet (10E9/L)	185.7 ± 133.9	194.1 ± 125.2	0.661
Hypersensitivity C reactive protein (mg/L)	100.5 ± 82.1	72.2 ± 58.4	0.02
Albumin (g/L)	31.8 ± 5	33.7 ± 7.3	0.037
Alanine transaminase (U/L)	25.5 (12–72)	12.00	0.607
Aspartate aminotransferase (U/L)	28.5 (12–75.8)	21.00	0.34
Cholinesterase (U/L)	3496.8 ± 1853.2	4648.5 ± 2368.3	<0.001
Total bilirubin (μmol/L)	15.5 (10–47)	15.5 (10–34)	0.457
Serum creatinine (μmol/L)	65 (47.8–113.8)	67.5 (52.3–92.8)	0.379
INR	1.1 (1.0–1.2)	1.0 (0.9–1.2)	0.54
