# Supplementary material for: Infections Caused by Carbapenemase-Producing Klebsiella pneumoniae: Microbiological Characteristics and Risk Factors
Source: Microb Drug Resist. 2019 Mar 8;25(2):287–96. doi: 10.1089/mdr.2018.0339 (PMC6441289; doi:10.1089/mdr.2018.0339)
Supplement: Supplemental data [file Supp_Table3.pdf]

Supplementary Table S3. Risk Factors for 28-Day Crude Mortality

	Univariate analysis			Multivariable analysis			
						95% CI for EXP(B)	
	Death (n=*27)*	*Survivors* (n=*171)*	p	*Sig.*	*Exp(B)*	*Lower*	*Upper*
Age (years)	59.1±14.0	57.6±15.2	0.632				
Gender, male, *n* (%)	18 (66.7)	115 (67.3)	0.952				
CPKP, *n* (%)	12 (44.4)	54 (31.6)	0.188				
APACHE II score	14.5±6.7	9.7±5.7	<0.001				
Pitt bacteremia score >4, *n* (%)	13 (48.1)	26 (15.2)	<0.001	0.006	3.803	1.470	9.840
ICU stay, *n* (%)	18 (66.7)	59 (34.5)	0.001				
K. pneumoniae identified as the first pathogens, *n* (%)	14 (51.9)	88 (51.5)	0.894				
Single pathogen, *n* (%)	8 (29.6)	67 (39.2)	0.342				
Concomitant G– infection, *n* (%)	14 (51.9)	75 (43.9)	0.438				
Concomitant G+ infection, *n* (%)	9 (33.3)	57 (33.3)	1				
Concomitant fungus infection, *n* (%)	7 (25.9)	43 (25.1)	0.931				
Metastatic infection, *n* (%)	8 (29.6)	31 (18.1)	0.163				
Bacteremia, *n* (%)	14 (51.9)	46 (26.9)	0.009				
Admission due to bacteremia, *n* (%)	6 (22.2)	12 (7.0)	0.011				
Admission due to sepsis, *n* (%)	6 (22.2)	17 (9.9)	0.064				
Invasive procedure or devices							
Surgery, *n* (%)	12 (44.4)	96 (56.1)	0.257				
Urinary catheterization, *n* (%)	22 (81.5)	120 (70.2)	0.225				
Venous catheterization, *n* (%)	27 (100)	168 (98.2)	0.488				
Arterial catheterization, *n* (%)	18 (66.7)	84 (49.1)	0.09				
Stomach tube, *n* (%)	22 (81.5)	83 (48.5)	0.001				
Mechanical ventilation, *n* (%)	19 (70.4)	58 (33.9)	<0.001				
Tracheotomy, *n* (%)	8 (29.6)	32 (18.7)	0.189				
Continuous renal replacement therapy, *n* (%)	9 (33.3)	22 (12.9)	0.007				
Hemodialysis, *n* (%)	6 (22.2)	30 (17.5)	0.558				
Bronchofibroscope use, *n* (%)	0	4 (2.3)	0.422				
Wound drainage tube use, *n* (%)	16 (59.3)	116 (68.7)	0.38				
Prior chemotherapy or radiotherapy, *n* (%)	2 (7.4)	12 (7.0)	0.941				
Prior corticosteroid therapy, *n* (%)	9 (33.3)	41 (24.0)	0.289				
Prior immunosuppressant use, *n* (%)	3 (11.1)	15 (8.8)	0.694				
Pre-existing medical conditions							
Diabetes, *n* (%)	4 (14.8)	32 (18.7)	0.625				
Hepatitis, *n* (%)	7 (25.9)	26 (15.2)	0.165				
Tumor, *n* (%)	8 (29.6)	51 (29.8)	0.984				
Hypertension, *n* (%)	9 (33.3)	48 (28.1)	0.575				
Coronary heart disease, *n* (%)	1 (3.7)	4 (2.3)	0.674				
Cerebral infarction, *n* (%)	0	3 (1.8)	0.488				
Renal insufficiency, *n* (%)	0	3 (1.8)	0.488				
Trauma, *n* (%)	1 (3.7)	1 (0.6)	0.132				
Organ transplant, *n* (%)	1 (3.7)	1 (0.6)	0.123				
Antimicrobial therapy after diagnosis							
β-lactam and/or β-lactamase inhibitor, *n* (%)	16 (59.3)	118 (69)	0.314				
Cephalosporins, *n* (%)	2 (7.4)	47 (27.5)	0.025	0.049	0.207	0.043	0.998
Carbapenems, *n* (%)	24 (88.9)	103 (60.2)	0.04				
Fluoroquinolone, *n* (%)	2 (7.4)	42 (24.6)	0.046				
Aminoglycoside, *n* (%)	4 (14.8)	12 (7.0)	0.167				
Vancomycin, *n* (%)	5 (18.5)	24 (14)	0.54				
Tigecycline, *n* (%)	8 (29.6)	26 (15.2)	0.065				
Teicoplanin, *n* (%)	1 (3.7)	30 (17.5)	0.066				
Ornidazole, *n* (%)	2 (7.4)	13 (7.6)	0.972				
Linezolid, *n* (%)	2 (7.4)	6 (3.5)	0.339				
Fosfomycin, *n* (%)	3 (11.1)	8 (4.7)	0.175				
Daptomycin, *n* (%)	3 (11.1)	1 (0.6)	<0.001				
Combination therapy, *n* (%)	16 (59.3)	85 (49.7)	0.356				
Laboratory examination							
White blood cell (10E9/L)	13.3±11.1	9.7±6.2	0.014				

(*continued*)

Supplementary Table S3. (Continued)

	Univariate analysis			*Multivariable analysis*			
						95% CI for EXP(B)	
	Death (n = *27)*	*Survivors* (n = *171)*	p	*Sig.*	*Exp(B)*	*Lower*	*Upper*
Neutrophil percentage (%)	86.9 ± 8.2	75.4 ± 16.4	<0.001	0.016	1.069	1.012	1.128
Hemoglobin (g/L)	102.7 ± 24.2	114.2 ± 90.2	0.512				
Platelet (10E9/L)	118 (40–175)	183 (116–255)	0.04				
Hypersensitivity C reactive protein (mg/L)	99.8 (57.6–227.1)	52.6 (15.1–106)	0.004				
Albumin (g/L)	28.6 ± 4.8	33.7 ± 6.7	<0.001	0.004	0.306	0.137	0.685
Alanine transaminase (U/L)	47 (19–97)	27 (12–72)	0.635				
Aspartate aminotransferase (U/L)	56 (24–100)	28 (18–63)	0.228				
Cholinesterase (U/L)	2640.5 ± 1056	4521 ± 2307.2	<0.001				
Total bilirubin (μmol/L)	34 (14–131)	15 (10–30)	0.927				
Serum creatinine (μmol/L)	79 (48–153)	67 (50–93)	0.62				
INR	1.2 (1.1–1.4)	1.1 (1–1.2)	0.975				
