# Supplementary material for: Infections Caused by Carbapenemase-Producing Klebsiella pneumoniae: Microbiological Characteristics and Risk Factors
Source: Microb Drug Resist. 2019 Mar 8;25(2):287–96. doi: 10.1089/mdr.2018.0339 (PMC6441289; doi:10.1089/mdr.2018.0339)
Supplement: Supplemental data [file Supp_Table4.pdf]

Supplementary Table S4. Risk Factors for Crude In-Hospital Mortality at Univariate Analysis

	Death (n = *62*)	*Survivors* (n = *136*)	p
Age (years)	60.7 ± 13.4	56.54 ± 15.6	0.07
Gender, male, *n* (%)	41 (66.1)	92 (67.6)	0.833
CPKP, *n* (%)	38 (61.3)	28 (20.6)	<0.001
APACHE II score	14.1 ± 5.9	8.6 ± 5.3	<0.001
Pitt bacteremia score >4, *n* (%)	38 (61.3)	15 (11.0)	<0.001
ICU stay, *n* (%)	40 (64.5)	37 (27.2)	<0.001
Hospital day (days)	36 (18.8–59.8)	27.5 (12.3–43.8)	<0.001
K. pneumoniae identified as the first pathogens, *n* (%)	19 (30.6)	83 (61)	0.001
Single pathogen, *n* (%)	9 (14.5)	66 (48.5)	<0.001
Concomitant G– infection, *n* (%)	40 (64.5)	49 (36.0)	<0.001
Concomitant G+ infection, *n* (%)	31 (50.0)	35 (25.7)	<0.001
Concomitant fungus infection, *n* (%)	27 (43.5)	23 (16.9)	0.01
Metastatic infection, *n* (%)	18 (29.0)	21 (15.4)	<0.001
Bacteremia, *n* (%)	36 (58.1)	24 (17.6)	0.026
Admission due to bacteremia, *n* (%)	7 (11.3)	11 (8.1)	<0.001
Admission due to sepsis, *n* (%)	12 (19.4)	11 (8.1)	0.467
Invasive procedure or devices			
Surgery, *n* (%)	28 (45.2)	80 (58.8)	0.022
Urinary catheterization, *n* (%)	52 (83.9)	90 (66.2)	0.073
Venous catheterization, *n* (%)	62 (100)	133 (97.8)	0.01
Arterial catheterization, *n* (%)	45 (72.6)	57 (41.9)	0.239
Stomach tube, *n* (%)	49 (79)	56 (41.2)	<0.001
Mechanical ventilation, *n* (%)	43 (69.4)	34 (25)	<0.001
Tracheotomy, *n* (%)	27 (43.5)	13 (9.6)	<0.001
Continuous renal replacement therapy, *n* (%)	22 (35.5)	9 (6.6)	<0.001
Hemodialysis, *n* (%)	22 (35.5)	14 (10.3)	0.035
Bronchofibroscope use, *n* (%)	1 (1.6)	3 (2.2)	<0.001
Wound drainage tube use, *n* (%)	42 (67.7)	90 (66.2)	0.783
Prior chemotherapy or radiotherapy, *n* (%)	5 (8.1)	9 (6.6)	0.828
Prior corticosteroid therapy, *n* (%)	25 (40.3)	25 (18.4)	0.713
Prior immunosuppressant use, *n* (%)	6 (9.7)	12 (8.8)	0.001
Pre-existing medical conditions			
Diabetes, *n* (%)	14 (22.6)	22 (16.2)	0.089
Hepatitis, *n* (%)	12 (19.4)	21 (15.4)	0.279
Tumor, *n* (%)	18 (29.0)	41 (30.1)	0.493
Hypertension, *n* (%)	24 (38.7)	33 (24.3)	0.874
Coronary heart disease, *n* (%)	3 (4.8)	2 (1.5)	0.037
Cerebral infarction, *n* (%)	3 (4.8)	0	0.161
Renal insufficiency, *n* (%)	1 (1.6)	2 (1.5)	0.01
Trauma, *n* (%)	2 (3.2)	0	0.939
Organ transplant, *n* (%)	2 (3.2)	0	0.035
Antimicrobial therapy after diagnosis			
β-lactam and/or β-lactamase inhibitor, *n* (%)	42 (67.7)	92 (67.6)	<0.001
Cephalosporins, *n* (%)	11 (17.7)	38 (27.9)	0.989
Carbapenems, *n* (%)	55 (88.7)	72 (52.9)	<0.001
Fluoroquinolone, *n* (%)	10 (16.1)	34 (25.0)	0.123
Aminoglycoside, *n* (%)	9 (14.5)	7 (5.1)	0.167
Vancomycin, *n* (%)	18 (29.0)	11 (8.1)	0.025
Tigecycline, *n* (%)	23 (37.1)	11 (8.1)	0.586
Teicoplanin, *n* (%)	11 (17.7)	20 (14.7)	<0.001
Ornidazole, *n* (%)	3 (4.8)	12 (8.8)	0.057
Linezolid, *n* (%)	4 (6.5)	4 (2.9)	<0.001
Fosfomycin, *n* (%)	5 (8.1)	6 (4.4)	0.245
Daptomycin, *n* (%)	3 (4.8)	1 (0.7)	0.298
Combination therapy, *n* (%)	43 (69.4)	58 (42.6)	0.326
Laboratory examination			
White blood cell (10E9/L)	11.9 ± 9.8	9.3 ± 5.4	<0.001
Neutrophil percentage (%)	82.7 ± 14.8	74.4 ± 15.9	0.054
Hemoglobin (g/L)	101.2 ± 27	117.8 ± 99.9	0.001
Platelet (10E9/L)	165.9 ± 120.0	202.9 ± 130.1	0.199
Hypersensitivity C reactive protein (mg/L)	105.7 ± 91.5	70.7 ± 72.8	0.059
Albumin (g/L)	31.0 ± 5.8	34.0 ± 6.9	0.004

(*continued*)

Supplementary Table S4. (Continued)

	Death (n = *62*)	*Survivors* (n = *136*)	p
Alanine transaminase (U/L)	40 (16–82.3)	26.5 (12–83)	0.003
Aspartate aminotransferase (U/L)	38 (21.8–75.8)	28 (17–61.3)	0.742
Cholinesterase (U/L)	3237.5 ± 1795.0	4732.8 ± 2316.8	0.071
Total bilirubin (μmol/L)	18 (10.8–61.8)	15 (10–29.8)	<0.001
Serum creatinine (μmol/L)	69.5 (47–132.3)	67 (51.3–91.8)	0.708
INR	1.1 (1–1.3)	1.0 (0.9–1.2)	0.912
